# Supplementary figures and images for: A Single-Cell Level and Connectome-Derived Computational Model of the Drosophila Brain
Source: Front Neuroinform. 2019 Jan 10;12:99. doi: 10.3389/fninf.2018.00099 (PMC6335393; doi:10.3389/fninf.2018.00099)

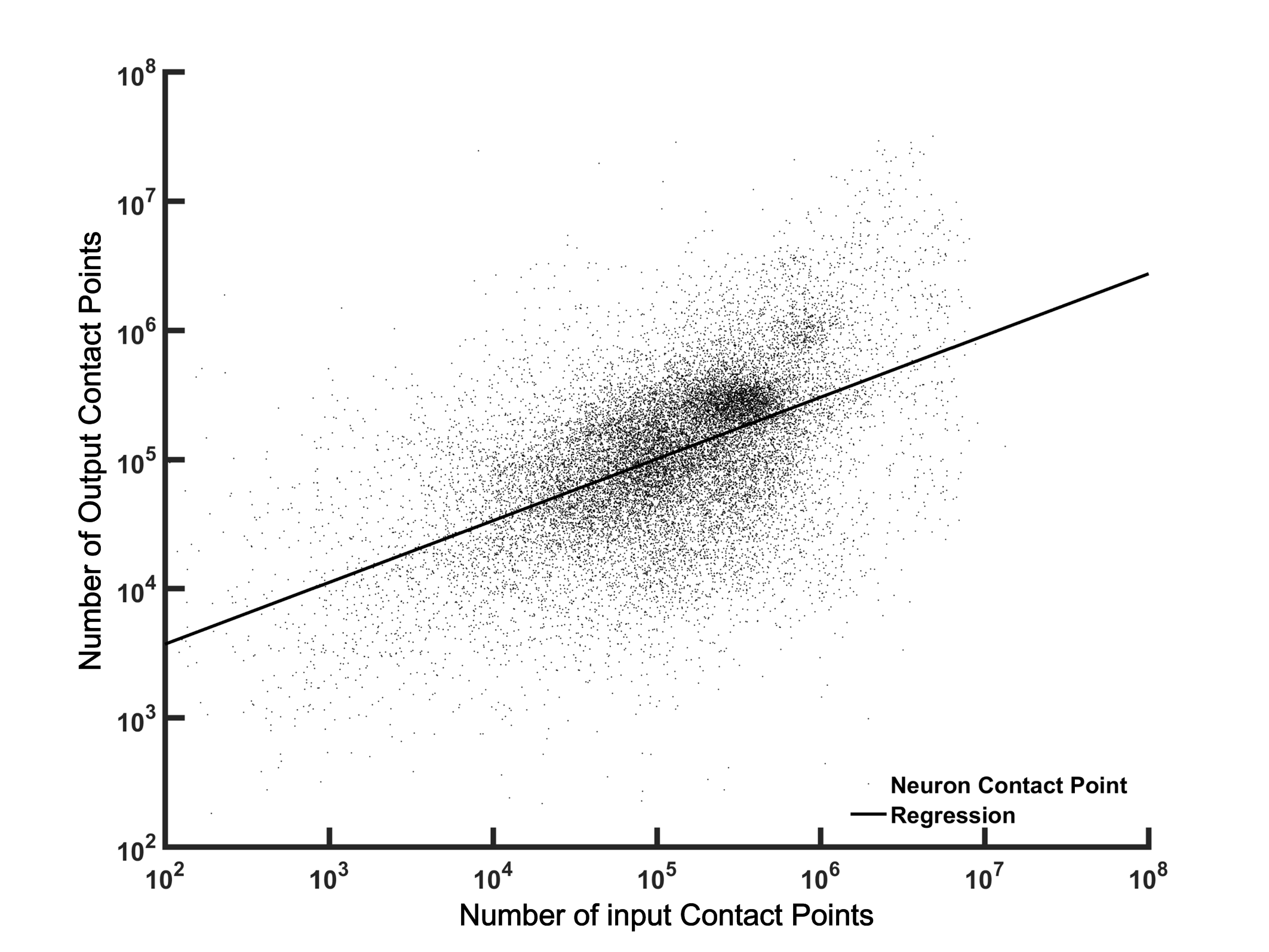

Supplement: Figure S1 — The input (ordinate) and output (abscissa) contact points of each neuron generally follow a linear relationship in a double-logarithmic plot. The solid line represents the linear regression of the data: log(y) = 0.48 * log(x) + 2.6. [file Data_Sheet_1.ZIP › figs/S1_Fig.tif]

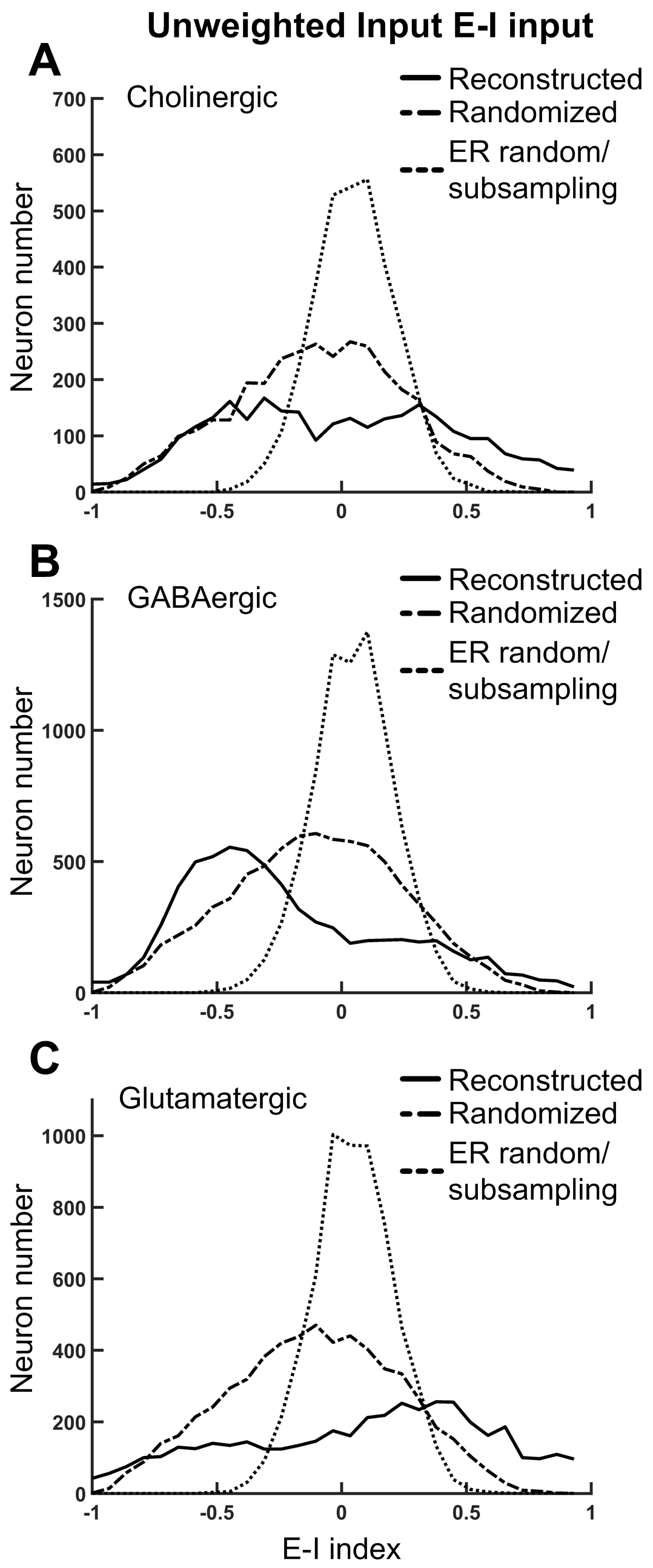

Supplement: Figure S1 — The input (ordinate) and output (abscissa) contact points of each neuron generally follow a linear relationship in a double-logarithmic plot. The solid line represents the linear regression of the data: log(y) = 0.48 * log(x) + 2.6. [file Data_Sheet_1.ZIP › figs/S2_Fig.tif]

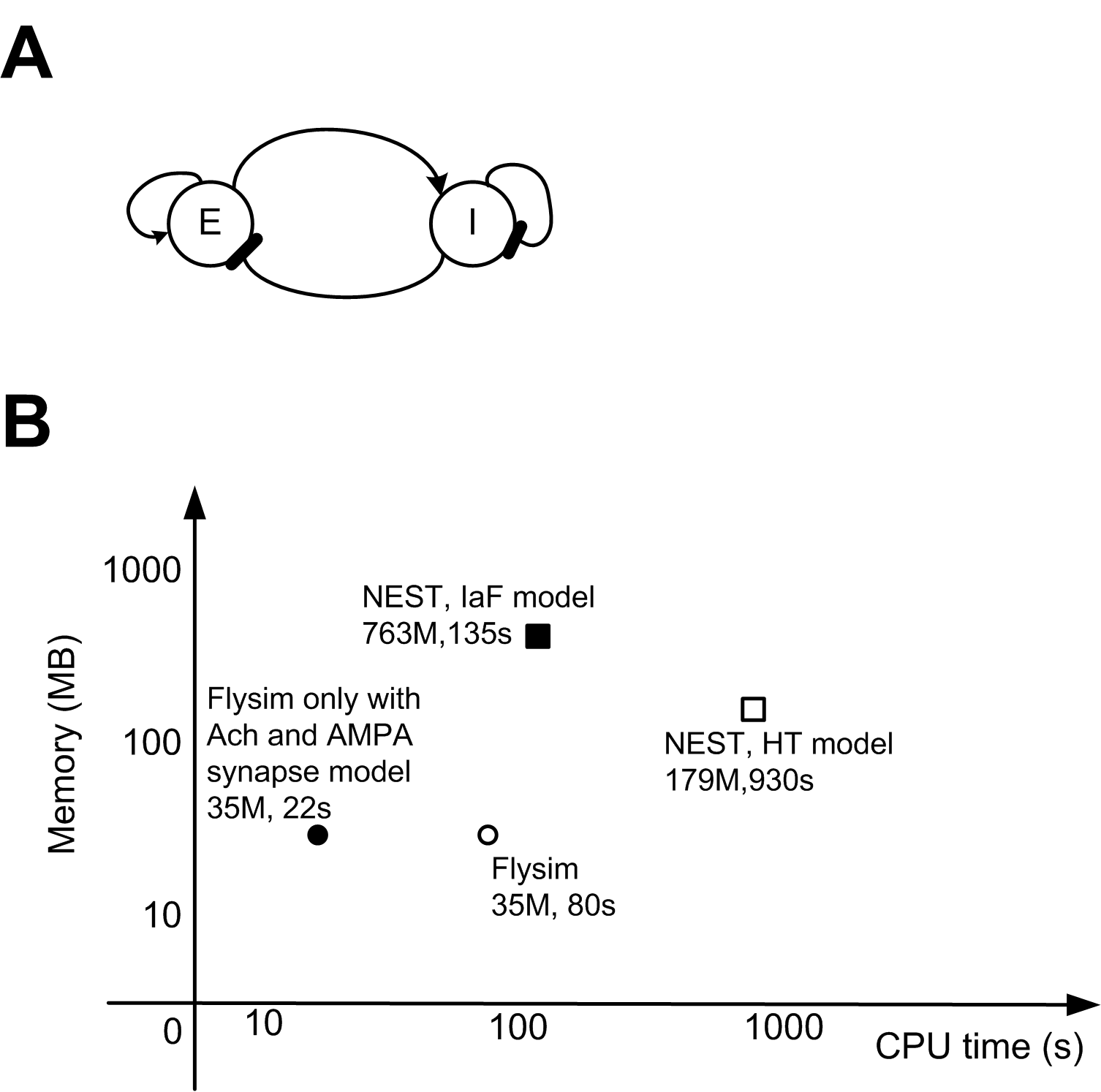

Supplement: Figure S1 — The input (ordinate) and output (abscissa) contact points of each neuron generally follow a linear relationship in a double-logarithmic plot. The solid line represents the linear regression of the data: log(y) = 0.48 * log(x) + 2.6. [file Data_Sheet_1.ZIP › figs/S3_Fig.tif]
